# Lack of Evidence for mtDNA as a Biomarker of Innate Immune Activation in HIV Infection

**DOI:** 10.1371/journal.pone.0050486

**Published:** 2012-11-29

**Authors:** Adam S. Lauring, Tzong-Hae Lee, Jeffrey N. Martin, Peter W. Hunt, Steven G. Deeks, Michael Busch

**Affiliations:** 1 Department of Medicine, University of California San Francisco, San Francisco, California, United States of America; 2 Department of Laboratory Medicine, University of California San Francisco, San Francisco, California, United States of America; 3 Blood Systems Research Institute, San Francisco, California, United States of America; University Hospital Zurich, Switzerland

## Abstract

Many human immunodeficiency virus (HIV) infected individuals suffer from persistent immune activation. Chronic inflammation and immune dysregulation have been associated with an increased risk of age-related diseases even among patients on highly active antiretroviral therapy. The factors leading to immune activation are complex, but have been hypothesized to include persistent viral replication with cellular death as well as microbial translocation across the gastrointestinal tract. Both processes may trigger innate immune responses since many native molecules released from dying cells are similar in structure to pathogen associated molecular patterns. These damage associated molecular patterns include mitochondrial DNA and formylated peptides. We hypothesized that circulating mitochondrial nucleic acid could serve as a biomarker for HIV-associated cell death and drive innate immune activation in infected individuals. We developed a quantitative polymerase chain reaction assay for plasma mitochondrial DNA and validated it on normal blood donors. We then measured mitochondrial DNA levels in acute and chronic HIV infection. While the assay proved to be accurate with a robust dynamic range, we did not find a significant association between HIV disease status and circulating mitochondrial DNA. We did, however, observe a negative correlation between age and plasma mitochondrial DNA levels in individuals with well-controlled HIV.

## Introduction

Persistent immune activation is a defining feature of HIV pathogenesis and progression to the acquired immune deficiency syndrome (AIDS). While early models focused on direct infection as a driver of CD4+ T cell depletion, it is clear that the majority of cell death during chronic infection is caused by indirect effects, including generalized T cell activation and apoptosis [1]. The activation phenotype affects the entire immune system and includes increased T cell activation [2], increased T cell turnover [3], polyclonal B cell activation [4], and increased levels of pro-inflammatory cytokines [5]. Immune activation is a significant predictor of disease progression in untreated persons [6], [7]. Elevated levels of T cell activation persist even after years of effective viral suppression from antiretroviral therapy [8], and appear to predict disease progression in these individuals [9]. T cell activation is also elevated in those rare individuals who are able to maintain durable control of HIV replication in the absence of therapy (“elite” controllers) and is associated with markers of mucosal damage and CD4+ T cell loss [10].

Systemic immune activation has also been invoked to explain the higher incidence of many chronic inflammatory conditions in people living with HIV [11]. Despite the effectiveness of antiretroviral therapy, HIV positive individuals have an elevated risk for heart, liver, kidney, and bone disease [12], and these risks are well correlated with markers of chronic immune activation. While viral suppression reduces the degree of T cell activation and general immune dysfunction, a chronic inflammatory state persists in many cases [13]. A clearer understanding of the HIV-associated inflammatory process and its relationship to end organ pathology will inform subsequent immune-directed therapeutic interventions [14].

While the effects of systemic immune activation are long-lasting, they appear to be driven in large part by events that occur in the first weeks following HIV infection [1]. Direct infection of CD4+ T cells in the gut-associated lymphoid tissue triggers profound alterations in mucosal immunity [15]. Studies of humans and other primates suggest that this early damage to the lymphoid and epithelial populations of the gastrointestinal tract leads to microbial translocation across the mucosal barrier. These microbes contain lipopolysaccharide and other pathogen associated molecular patterns (PAMPs) that are recognized by cells of the innate immune system [16]. The pro-inflammatory cytokines released by these cells prime further T cell activation, initiating a positive feedback loop of mucosal damage and immune dysfunction.

Like PAMPs, some cellular molecules serve as potent stimuli for innate immune responses. These damage associated molecular patterns, or DAMPs, are released from cells during tissue injury. They are often recognized by the same pattern recognition receptors as PAMPs and initiate a non-infectious inflammatory response [17]. For example, the chromatin factor HMGB1 is recognized by the receptor for glycated end-products (RAGE) and may potentiate the end organ effects of shock after trauma or sepsis [18]–[20]. As a result of their endosymbiotic origin, mitochondria contain several DAMPs [21]. Their formylated peptides are bound by formyl peptide receptor 1 on neutrophils, and the CpG repeats of mitochondrial DNA (mtDNA) are similarly recognized by TLR9. Recent work indicates that release of these mitochondrial DAMPS during trauma causes a severe inflammatory response, indistinguishable from sepsis [22].

We hypothesized that mitochondrial DNA might be elevated in HIV infected adults and contribute to the immunopathogenesis of HIV-related disease. In this model, plasma levels of mtDNA would provide a read-out of cellular death over time. As a DAMP, mtDNA could also contribute to the pro-inflammatory milieu characteristic of HIV-infection. We tested our hypothesis by measuring mtDNA levels in fractionated plasma during acute and chronic infection. While we found no clear association between mtDNA and HIV disease state, we did observe a significant negative correlation between age and mtDNA.

## Materials and Methods

### Study Participants and Plasma Preparation

Adults with acute HIV infection were sampled from plasma donors with incident seroconversion [23]–[25]. Plasma donations (600–800 ml) from source plasma donors were routinely collected at approximately twice weekly intervals and stored frozen at −20°C or less. After confirmation of anti-HIV or p24 antigen seroconversion, donors were permanently deferred from further donation, and all quarantined plasma donations, i.e. donations routinely held back from distribution for a 60-day waiting period, were retrieved from storage to construct panels containing sequentially drawn plasma samples from seroconverting donors. Each donation was rapidly thawed, aliquoted, and the aliquots re-frozen at −20°C or less. Serial donation aliquots were coded and compiled into anonymized panels, not linked to individual donors. Records of each donor visit date, as well as the results of routine and research laboratory tests for each plasma aliquot, were entered in a computerized database. Panel samples were obtained from Alpha Therapeutic Corporation (Los Angeles, CA, USA) and Boston Biomedica (West Bridgewater, MA, USA). These panels have been previously utilized in a series of studies characterizing the kinetics of viremia and immune responses in early HIV infection [23]–[25].

Adults with chronic HIV infection were sampled from a clinic-based cohort of over 1500 chronically HIV-infected and uninfected individuals at the University of California, San Francisco (SCOPE). Participants are followed every four months with detailed questionnaires, clinical laboratory monitoring, and biological specimen banking. From this cohort, we evaluated three distinct groups of HIV-infected individuals: (1) “Elite controllers”, defined as HIV-seropositive individuals maintaining plasma HIV RNA levels <75 copies/ml in the absence of therapy (episodes of clinically detectable viremia in the previous year were allowed if they were followed by undetectable values); (2) “HAART-suppressed” individuals maintaining plasma HIV RNA levels <75 copies/ml on antiretroviral therapy spanning at least 3 months prior to specimen date and including date of specimen; and (3) untreated HIV “non-controllers” with plasma HIV RNA levels above 10,000 copies/ml. HIV negative individuals were enrolled as controls as part of this cohort. Plasma from SCOPE participants were processed by the AIDS Specimen Bank at UCSF. Briefly, vacutainer tubes were spun at 1000 g for 10 minutes, with the brakes off. The plasma layers were transferred into a fresh conical tube and spun at 1000 g for 10 minutes, brakes on. Aliquots of these clarified plasma samples were stored at −70°C until use.

All participants provided written informed consent and this research was approved by the institutional review board of the University of California, San Francisco.

### Standardization with Platelet Rich Plasma

The platelet rich plasma used for the validation experiment was derived from a single blood donor. Three EDTA tubes were drawn and left to settle for one hour. The platelet rich plasma supernatant was removed, and the platelet count was determined by Sysmex XE-2100D (Kobe, Japan). This sample was spun at 3000 g for 5 minutes. The platelet pellet was digested in 100 µl of a 1∶1 mixture of Solution A (0.1 M KCl, 0.01 M Tris Base, 0.0025 M MgCl_2_*6H2O, pH 8.3) and Solution B (10 mM Tris pH 8.3, 2.5 mM MgCl_2_*6H_2_O, 1% Tween-20, 1% NP-40) supplemented with 12.5 µg proteinase K (Gibco BRL, Carlsbad, California). Following protein digestion at 60°C for 2 hours, proteinase K was inactivated by incubation at 100°C for 30 minutes. This lysate was serially diluted, and the mtDNA content in 5 µl of each sample determined as above.

### Plasma Fractionation and DNA Extraction

**Figure 1:**
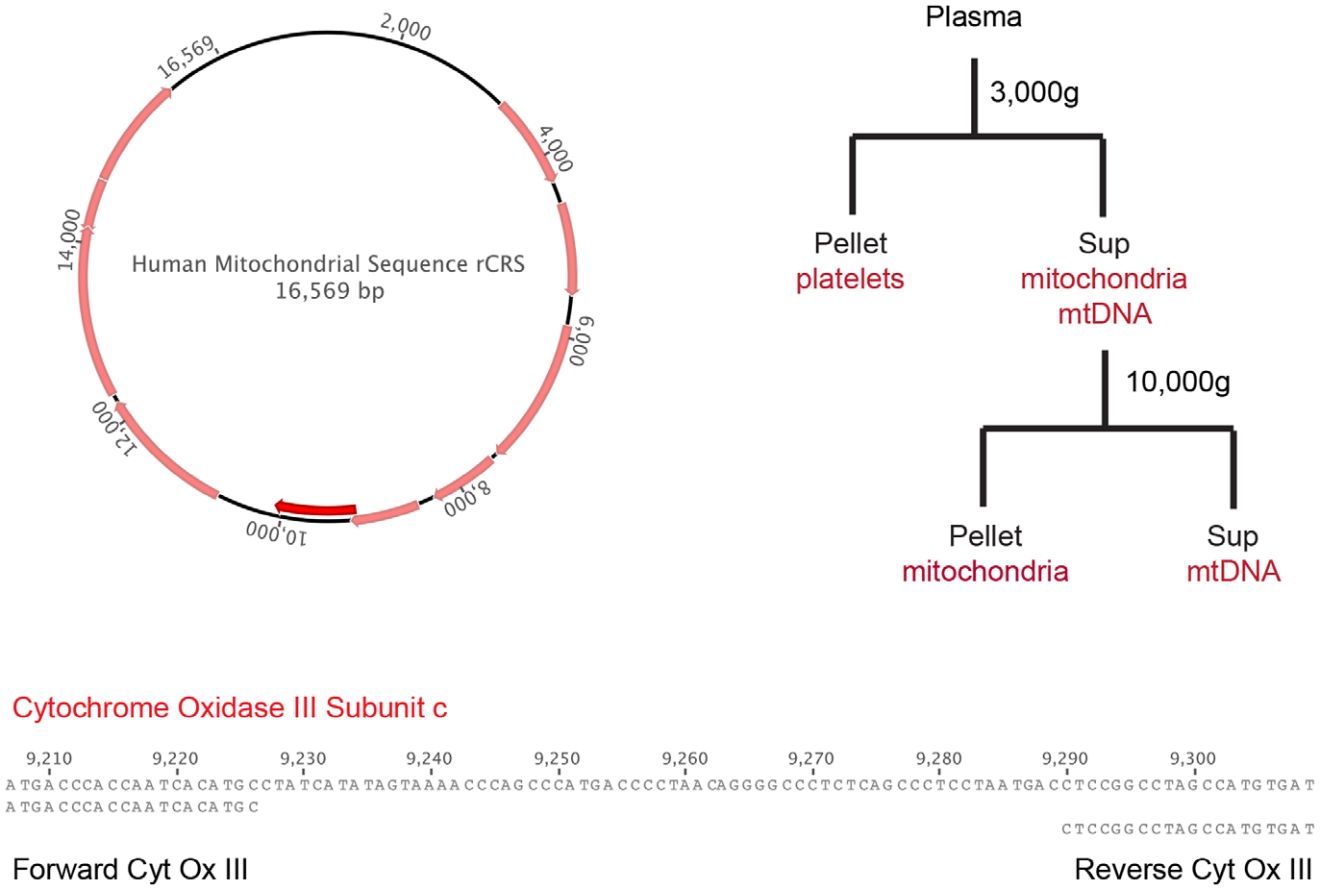
A real-time polymerase chain reaction assay for mtDNA. The circular mitochondrial genome is shown with the open reading frame for cytochrome oxidase III, subunit c indicated in bold. Below are the 100 bp PCR amplicon and the primers used in this study. The branching diagram shows the fractionation scheme used to derive fractions free of platelets (3000 g supernatant) and intact mitochondria (10,000 g supernatant) from donor plasma.

Plasma samples were fractionated as described in Figure 1. The 3000 g spin was for 5 minutes and the 10,000 g spin was for 30 minutes at 4°C, both in a microcentrifuge. All DNA extractions were performed using Qiagen 96 well kits. DNA was extracted from 50 µl plasma, 45 µl of 3000 g supernatant, or 40 µl of 10,000 g supernatant and eluted in 180 µl water. The eluates were adjusted to a 200 µl total volume of 1× solution A and B. For experiments examining the effect of freeze-thaw, each plasma fraction was subjected to repeated freeze-thaw cycles prior to DNA extraction.

### Quantitative PCR

Real time PCR reactions were performed in triplicate for each sample, with 5 µl of template in a 10 µl reaction. Each reaction contained 5 mM dNTPs, 5 mM MgCl_2_, 0.75 units FastStart Taq (Roche Diagnostics, Indianapolis, IN), 1 µM primer CoxF (5′ ATGACCCACCAATCACATGC 3′) and 1 µM CoxR (5′ ATCACATGGCTAGGCCGGAG 3′) (Integrated DNA Technologies, Coralville, IA), 0.375× SYBR Green (FMC BioProducts, Rockland, ME). The PCR program had an initial activation of 95°C/1 m; followed by 45 cycles of 95°C/30 s, 60°C/30 s, 72°C/45 s. Cycle thresholds (Ct) were determined by SYBR green fluorescence. These values were converted to absolute copy number based on a standard curve of spectrophotometer-determined copy number vs. Ct. All data are expressed as copy number per 5 µl template. This corresponds to approximately 1.25 µl plasma or its fractionated equivalent.

### Statistical Methods

Continuous variables were compared by Wilcoxon rank sum tests for pairwise comparisons. Relationships between continuous variables were assessed with Spearman’s rank order correlation coefficients. All statistical analysis was performed using R.

## Results

### Development of a Quantitative Assay for mtDNA

**Figure 2:**
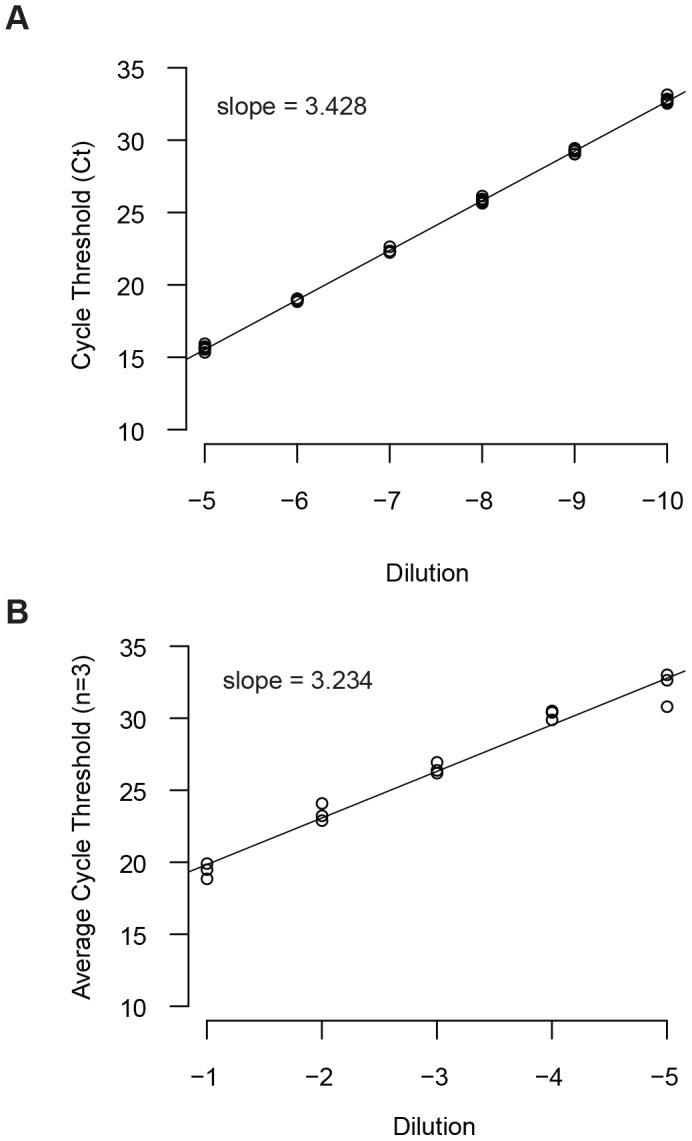
Sensitivity and dynamic range of qPCR assay for mitochondrial DNA. (A) Dynamic range of qPCR assay for mtDNA. A PCR amplicon containing the target region of cytochrome oxidase III, subunit c was serially diluted and detected using a SYBR green qPCR assay. Cycle threshold values (y-axis) for each dilution (x-axis) are shown for 3 technical replicates per dilution. The slope of the standard curve is shown at top-left. (B) The mtDNA qPCR assay can detect a single copy of mtDNA. A platelet pellet of 4.5*10^5^ was derived from donor plasma. A lysate of this pellet was serially diluted and the relative mtDNA content determined by qPCR for three technical replicates per dilution. Cycle threshold values (y-axis), dilution (x-axis), and the slope of the standard curve (top left) are shown. The assay was linear over a 5 log dilution series, or down to roughly one platelet and its associated mitochondrion.

The mitochondrial fraction of the human genome harbors significantly more genetic variation than the much larger nuclear component. Nevertheless, many mitochondrial genes have highly conserved regions. We based our quantitative PCR assay on one developed for a similar study [22], and designed primers that bound within the gene encoding cytochrome oxidase c, subunit III (Figure 1). While single nucleotide polymorphisms have been identified in the primer binding sites, none have been found at a frequency greater than 5%. These primers were highly efficient and we could easily measure the resulting 100 bp PCR product over a 5 log dynamic range using a SYBR green based assay (Figure 2A). We validated the sensitivity of our assay using platelet rich plasma derived from a healthy blood donor. The 3000 g pellet of this pooled plasma contained approximately 5*10^5^ platelets. When we measured the mtDNA content of this pellet, we were able to detect a signal at a 10^−5^ dilution, corresponding to roughly one platelet (Figure 2B). Since each platelet contains just 1–2 mitochondria [26], [27], this signal corresponds to a single copy of mtDNA.

**Figure 3:**
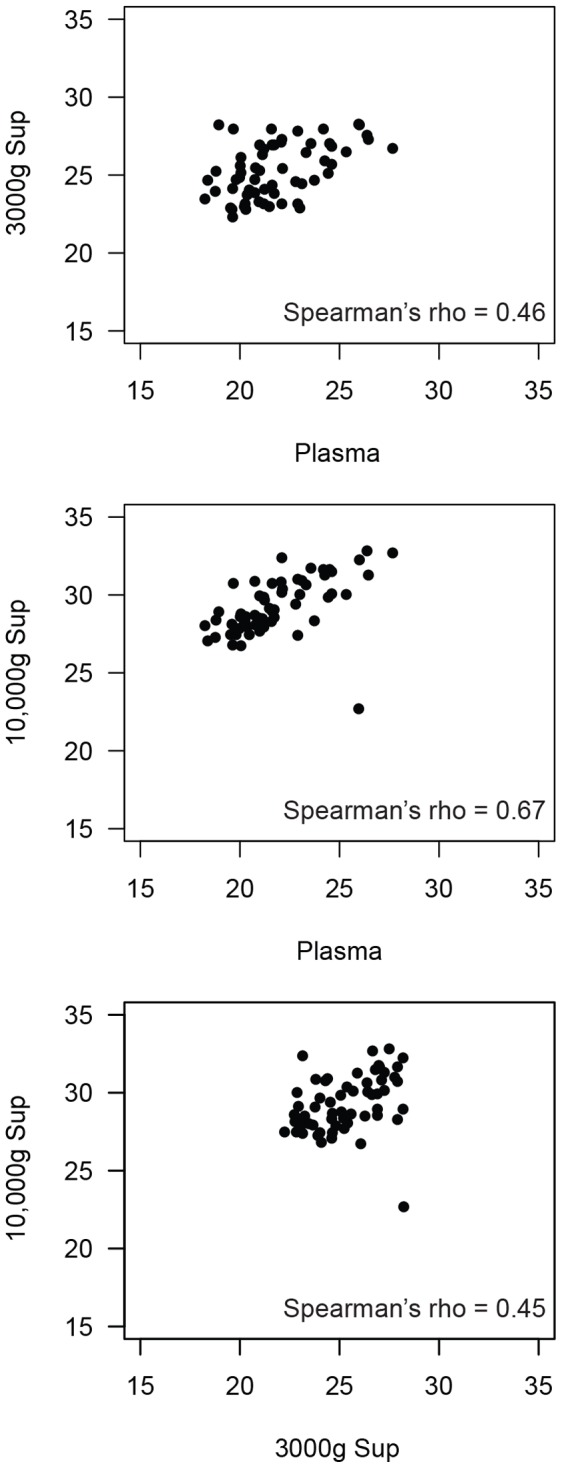
Comparison of mtDNA levels in plasma fractions. Three thousand and 10,000 g supernatants were derived from the plasma of 20 individuals. Correlation plots of mtDNA measurements (cycle threshold, x- and y-axes) are shown for plasma vs. 3000 g supernatant (top), plasma vs. 10,000 g supernatant (middle), and 3000 g vs. 10,000 g supernatants (bottom). Spearman’s rho for each comparison is shown at bottom-right.

Based on our experience with pathogen-directed real time PCR assays, we initially developed our assay for plasma samples. However, in healthy blood donors, we noticed that mtDNA levels were similar in serum and plasma. Because the clotting process generally results in spontaneous lysis of entrapped platelets and release of their mitochondria, we sought to determine whether residual platelets in these processed samples would lead to spurious mtDNA signal. We therefore used differential centrifugation to fractionate donor plasma and compared the mtDNA levels across multiple samples. We separated platelets from free mitochondria and mtDNA with a single 3000 g spin (Figure 1). This supernatant was then spun at 10,000 g to pellet intact mitochondria from the soluble fraction. In a pilot study of 20 HIV positive and negative individuals, we found good correlation among the fractions. The mtDNA signal was highest in the plasma and lowest in the 10,000 g supernatant. These data suggest that the low speed spin successfully removed residual platelets (and their mitochondria) from plasma, while the high-speed spin effectively separated intact mitochondria from free mtDNA (Figure 3). We found that the plasma and 10,000 g supernatant were highly correlated (Spearman’s rho = 0.67, p<0.0001), suggesting that measurements of plasma would reflect the amount of free mtDNA in our samples. Because the time and expense of the 10,000 g spin significantly reduced the number of samples we could study, we analyzed only plasma and 3000 g supernatants in our experiments.

**Figure 4:**
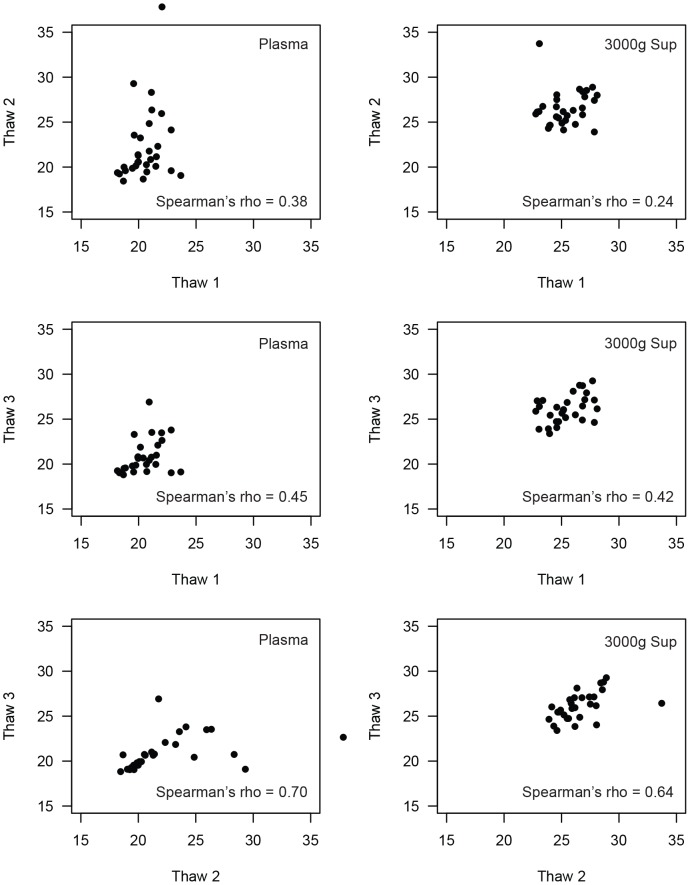
The effect of freeze-thaw cycles on mtDNA levels in plasma fractions. Plasma aliquots and 3000 g supernatants were prepared from 30 individuals and assayed fresh (Thaw 1) or after an additional one (Thaw 2) or two (Thaw 3) freeze-thaw cycles. Correlation plots of mtDNA measurements (cycle threshold, x- and y-axes) are shown for plasma (left) or 3000 g supernatant (right). Spearman’s rho for each comparison is shown at bottom-right.

Our goal was to use this assay to study mtDNA in both fresh and archived samples. Given the potential for spurious release of nucleic acid from lysed platelets, we examined the effect of multiple freeze-thaw cycles on mtDNA levels. As above, we used plasma and plasma-derived fractions from 30 HIV positive and 30 negative individuals. In general, we found agreement in mtDNA measurements between frozen samples measured after the initial thaw and those subjected to an additional freeze-thaw cycles (Figure 4). There were a number of outliers in both the plasma and 3000 g supernatant samples, resulting in generally low correlation coefficients (Spearman’s rho = 0.38 for plasma, rho = 0.24 for 3000 g supernatant). The correlation between samples subjected to two and three freeze-thaw cycles was much better for both fractions (Spearman’s rho = 0.70 for plasma, rho = 0.64 for 3000 g supernatant). These data indicate that multiple freeze-thaw cycles can potentially lead to biased results in mtDNA measurement. As a result, we used fresh samples prepared from the initial thaw whenever possible. For archived samples, we identified aliquots with a minimal number of freeze-thaw cycles and compared samples with a similar freeze-thaw history where possible.

**Figure 5:**
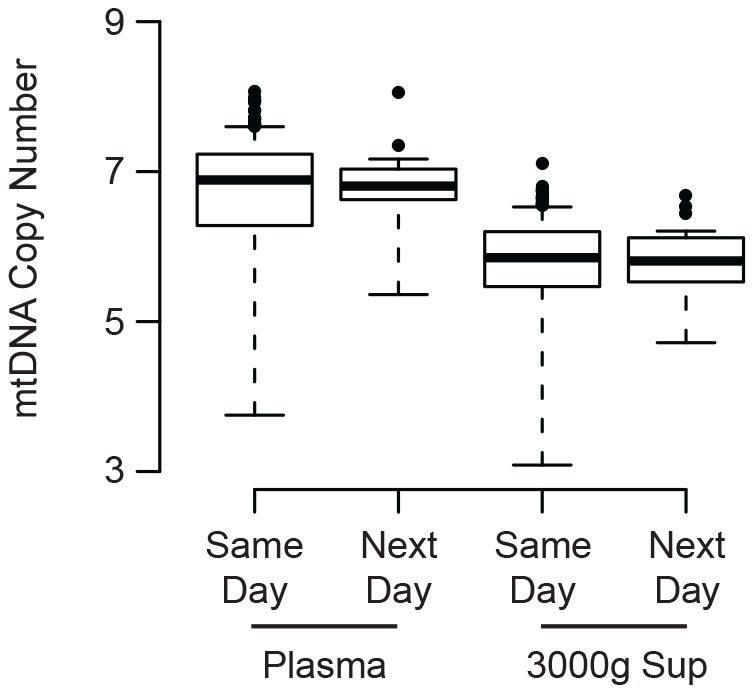
The effect of initial processing time on subsequent mtDNA measurements. Plasma from study participants was processed and frozen on either the same day (n = 385) or following morning (Next Day, n = 25). Plasma and 3000 g supernatants were processed from these frozen samples. Boxplots (median level, 25% and 75% quartiles, and 1.5 * interquartile range) show the mtDNA level (copy number, y-axis) for each fraction.

The vast majority (n = 410) of our samples came from individuals enrolled in a prospective cohort of HIV infected and uninfected adults (SCOPE). Phlebotomy, plasma preparation, and freezing were performed on the same day for most of these samples (n = 385). However, due to the timing of phlebotomy, a small minority (n = 25) were processed on the following day. We therefore examined whether delayed processing would bias mtDNA measurements in any way. Regardless of the time to processing, all plasma and 3000 g supernatants were derived from frozen aliquots on the initial thaw. We found that a short delay in processing did not significantly affect mtDNA levels in either plasma or the 3000 g supernatant fractions (Figure 5). In experiments with healthy blood donors, we did observe higher mtDNA levels in plasma samples that were derived from whole blood stored at room temperature for greater than 24 hours prior to processing (data not shown). This difference could suggest spurious release of mtDNA from cell breakdown over time. Overall, these data suggest that our mtDNA assay is reasonably robust to the heterogeneity in plasma processing times characteristic of many large clinical studies.

### Mitochondrial DNA Levels during Acute HIV Infection

**Figure 6:**
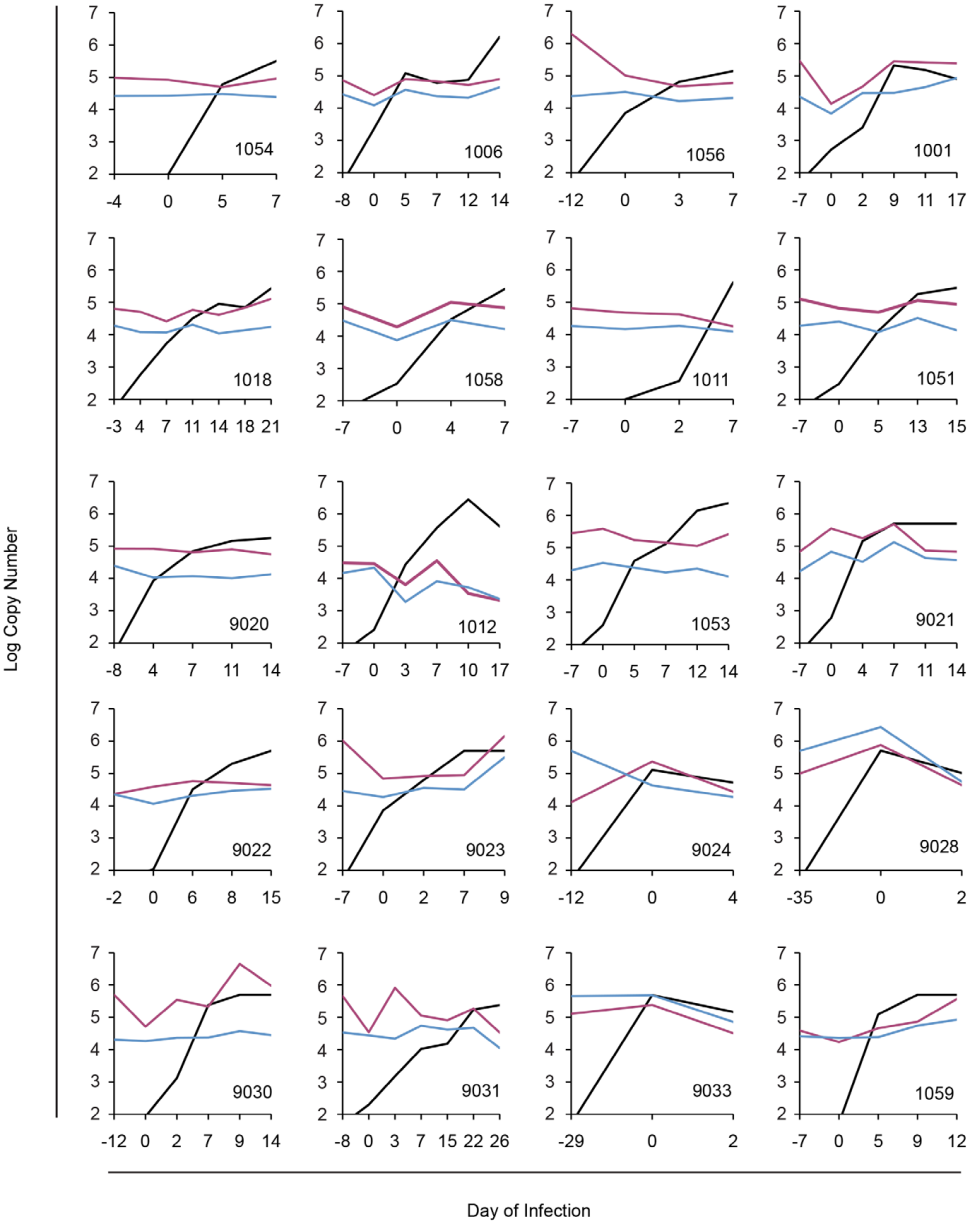
Mitochondrial DNA levels during acute HIV infection. Plasma and 3000 g supernatant fractions were derived from archived samples of plasma donors. Each plot shows results from a single donor with the day of infection (x-axis) indicating the onset of detectable viremia. Plasma viral load (black) and levels of mtDNA in plasma (red) and 3000 g supernatant (blue) are shown for each panel (subject code in lower right corner).

Given the massive cell death that is believed to occur in lymphoid tissues during acute HIV infection, we examined whether release of DAMPs from these cells would lead to a similar rise in plasma mtDNA levels. We tested a panel of archived longitudinal samples from 20 plasma donors, whose serial donations spanned the onset of detectable viremia. In nearly all cases, these samples were drawn during Fiebig Stages I-IV [24]. In the pre-viremic samples, we could easily detect mtDNA from the plasma and 3000 g fractions in all subjects (Figure 6). The mtDNA levels were generally similar to those of healthy donors (data not shown), and likely reflect each individual’s steady state. Over the time courses examined, we did not see a significant trend in plasma mtDNA levels across subjects; levels were event stable during those time points in which viremia peaked to over 100,000 copies/ml.

### Cross Sectional Analysis of DAMPS in Chronic HIV Infection

We also measured mtDNA in a cohort of individuals with chronic HIV infection, where the cumulative impact of HIV replication and/or HIV-associated immune dysfunction on plasma DAMP levels might be more apparent. We performed a cross-sectional analysis, measuring mtDNA in a single time point in untreated non-controllers, untreated controllers, HAART suppressed individuals, and HIV-negative individuals.

**Figure 7:**
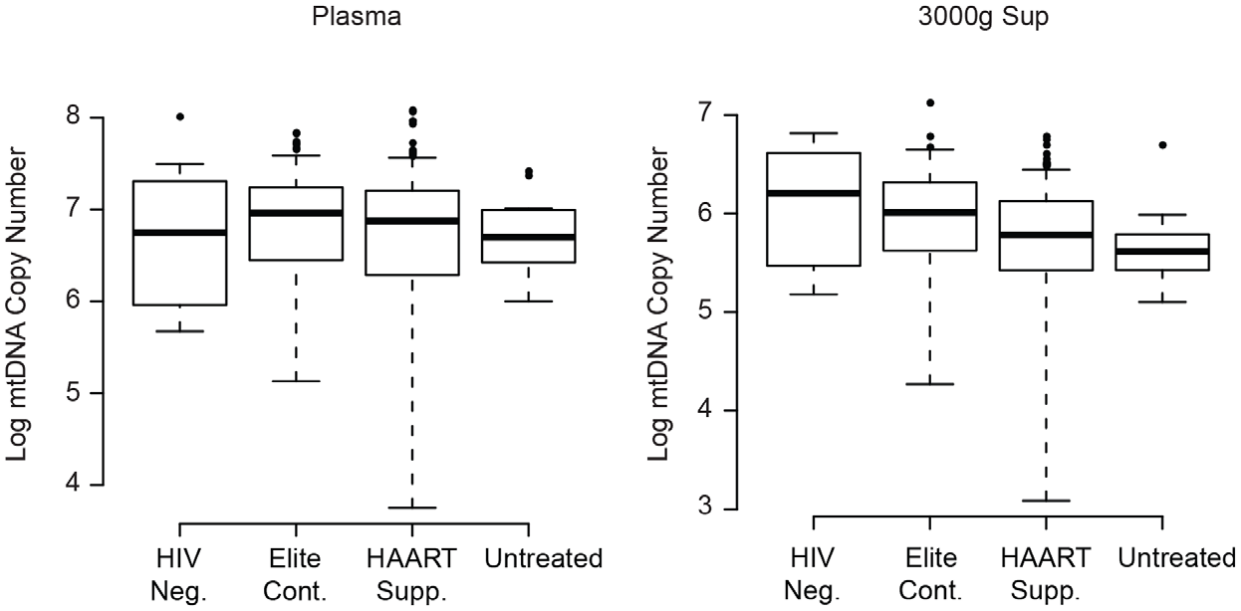
Mitochondrial DNA levels in chronic HIV infection. Mitochondrial DNA levels were measured in plasma (left) and 3000 g supernatant (right) from untreated, HIV-negative, HAART-suppressed and elite controller individuals (group definitions in Methods). Boxplots (median level, 25% and 75% quartiles, and 1.5 * interquartile range) show the mtDNA level (copy number, y-axis) for each fraction. P values are reported in the text.

Among these groups, we found little difference in plasma mtDNA levels (Figure 7). Most importantly, we found no statistically significant difference between the HIV-negative subjects and untreated, viremic individuals (p = 0.79 for plasma, p = 0.10 for 3000 g sup; Wilcoxon rank sum test). We also found no difference between the HIV-negative group and either elite controllers (p = 0.35 for plasma, p = 0.30 for 3000 g sup) or individuals whose viremia was adequately suppressed by HAART (p = 0.85 for plasma). The HAART-suppressed group exhibited lower mtDNA levels that the HIV-negative group within the 3000 g supernatant fraction (p = 0.01). We also observed no consistent trends among the HIV-positive subgroups. Plasma mtDNA levels were higher in the elite controllers compared to untreated individuals (p = 0.29 for plasma, p = 0.02 for 3000 g sup). Similarly, we found that mtDNA levels tended to be higher in HAART-suppressed individuals relative to those with untreated viremia (p = 0.68 for plasma, p = 0.30 for 3000 g sup). Only the difference in 3000 g supernatant levels between the elite controllers and untreated subgroup achieved statistical significance. The trend across all of the groups was for higher mtDNA levels in individuals with less ongoing viral replication.

### The Effect of Antiretroviral Medication on Plasma mtDNA Levels

**Figure 8:**
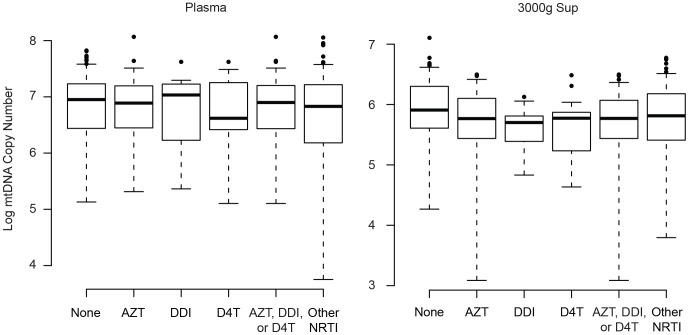
The nucleoside backbone does not influence plasma mtDNA. Mitochondrial DNA levels were measured in plasma (left) and 3000 g supernatant (right) from either untreated individuals (None) or subjects taking the indicated nucleoside analogues at the time of phlebotomy (x-axis). Boxplots (median level, 25% and 75% quartiles, and 1.5 * interquartile range) show the mtDNA level (copy number, y-axis) for each fraction. P values are reported in the text.

Several nucleoside reverse transcriptase inhibitors (NRTI) have off-target effects on mtDNA replication with complex effects on mitochondria in peripheral blood mononuclear cells. To further investigate this possibility, we compared mtDNA levels among HAART-suppressed individuals with different nucleoside regimens. Specifically, we compared those taking a NRTI with known mitochondrial toxicity (“AZT, ddI, or d4T”, Figure 8) to those with a non-toxic nucleoside backbone (“Other NRTI”). We found no difference between these groups in circulating mtDNA within either the plasma (p = 0.39) or 3000 g supernatant fractions (p = 0.41). We also found no statistically significant difference in our assays when we looked at each NRTI individually. Compared to the non-toxic NRTI group (“Other NRTI”), we found similar levels of mtDNA for the AZT (p = 0.44 for plasma, p = 0.66 for 3000 g sup), ddI (p = 0.62 for plasma, p = 0.22 for 3000 g sup), and d4T (p = 0.92 for plasma, p = 0.25 for 3000 g sup) subgroups. These data indicate that NRTI toxicity does not significantly influence total plasma mtDNA levels and is unlikely to confound our primary analysis.

### Plasma DAMPS Levels in HIV/HCV Coinfection

**Figure 9:**
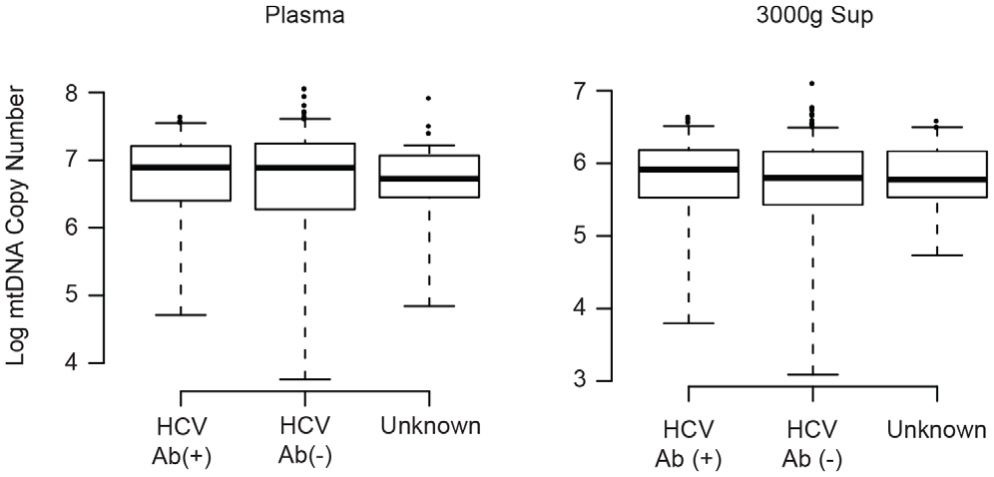
Hepatitis C virus coinfection does not influence plasma mtDNA levels in HIV infected individuals. Mitochondrial DNA levels were measured in plasma (left) and 3000 g supernatant (right) from either HCV coinfected (HCV Ab+) or HCV negative (HCV Ab-) subjects (x-axis). Boxplots (median level, 25% and 75% quartiles, and 1.5 * interquartile range) show the mtDNA level (copy number, y-axis) for each fraction. P values are reported in the text.

Our primary hypothesis was that active HIV replication and associated cell death would lead to release of cellular DAMPs, leading to increased levels of mtDNA in plasma. In this model, concurrent chronic infection and cytotoxicity could contribute to the aggregate mtDNA signal. Because many of our HIV-positive subjects were also chronically infected with hepatitis C, we asked whether coinfection confounded our results (Figure 9). We found that HCV serostatus did not influence mtDNA levels in either plasma (p = 0.97) or 3000 g supernatant (p = 0.30) fractions. While we did not have treatment or HCV viral load data for these individuals, these data indicate that coinfection did not contribute to the overall DAMP signal in a way that would bias our results. They further suggest that chronic viral infection alone does not contribute significantly to plasma mtDNA levels.

### Plasma mtDNA in HIV-infected Individuals Vary with Age

**Figure 10:**
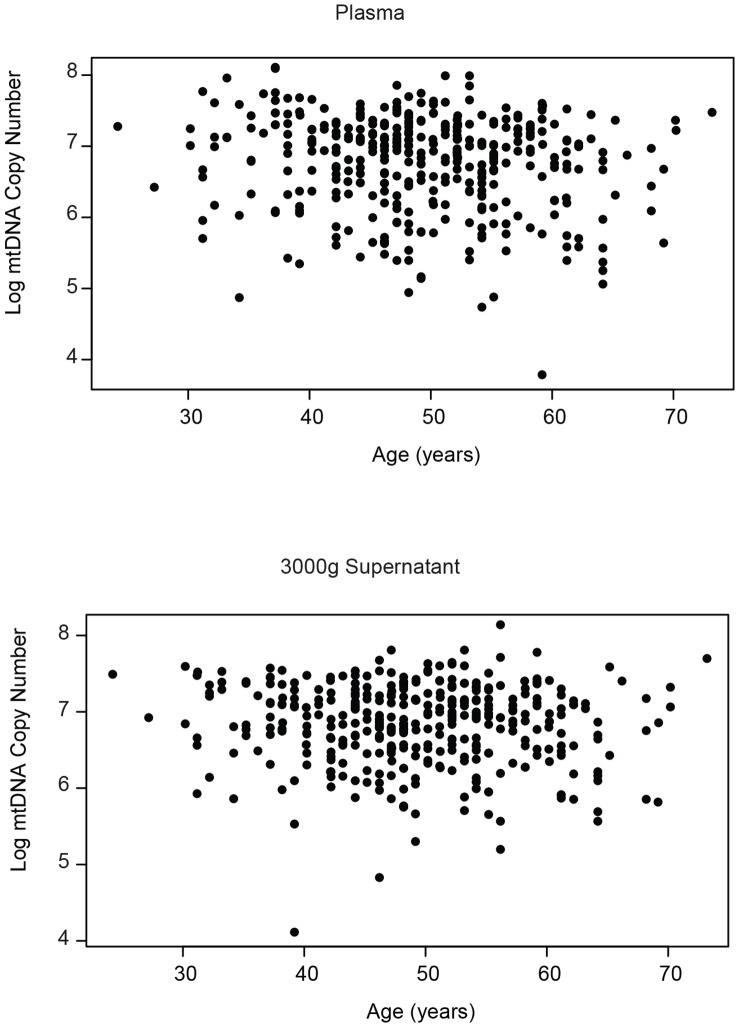
Age and plasma mtDNA levels are negatively correlated. Mitochondrial DNA levels were measured in plasma (top) and 3000 g supernatant (bottom) from HIV positive individuals with negative viral loads. This group (n = 374) included elite controllers and individuals on HAART. Scatterplots show the correlation between log mtDNA level (y-axis) and age (x-axis). Correlation coefficients and p values are reported in the text.

It is increasingly clear that individuals with HIV may be at higher risk for many age-associated, inflammatory diseases. While many social and epidemiologic factors contribute to this risk, irreversible HIV-associated immune dysfunction is thought to play a significant role. We therefore examined whether circulating mtDNA, a hypothesized DAMP, varied with age in our HIV-positive cohort (Figure 10). In a combined cohort (n = 374), including both elite controllers and HAART suppressed individuals, we found that plasma mtDNA levels decreased with age (Spearman’s rho = −0.13, p = 0.008). This correlation was driven largely by individuals on HAART (Spearman’s rho = −0.18, p = 0.003) as opposed to the elite controllers (Spearman’s rho = −0.03, p = 0.74). The correlation was less significant in the 3000 g supernatant fraction (Spearman’s rho = −0.07, p = 0.2253 for the combined cohort). These data suggest that plasma mtDNA levels could be a useful biomarker for age-related changes in HIV infected individuals.

## Discussion

Systemic immune activation in HIV infection is a complex process that involves multiple signaling pathways and affects all aspects of the immune system. We investigated whether mtDNA would be a useful biomarker for HIV-associated immune dysfunction. We hypothesized that plasma mtDNA would reflect the degree of cell death, and as a host cell DAMP, contribute to the pro-inflammatory immune response in acute and chronic infection. The quantitative PCR assay we developed was rigorously optimized to detect cell-free mitochondria and circulating mtDNA in freshly frozen or archived samples. During acute infection, we found no significant change in mtDNA levels in serial samples from 20 individuals. In a cross-sectional study, we found no difference among untreated, elite controller, HAART-suppressed, or HIV negative subgroups. Similarly, we found no difference when infected individuals were grouped according HCV serostatus or antiretroviral regimen. Interestingly, we did identify a negative correlation between age and plasma mtDNA among individuals on effective HAART.

While our data suggest that HIV infection has no impact on mtDNA levels, several issues make us cautious in interpreting these results. As in all negative studies, there is a possibility of type II error. Interestingly, our initial pilot studies of HIV-infected individuals suggested a significant association between plasma mtDNA and HIV status, with only 20 individuals in each group. We expected a similar result in the larger cross-sectional study of 410 individuals presented here. Despite the study’s larger size, our statistical power was only marginally increased since the HIV negative (n = 24) and untreated (n = 12) groups remained small. We also found significant variability in the HIV negative group. Together with the surprisingly high baseline mtDNA levels in all individuals, this variability could make it difficult to distinguish subtle, but statistically significant differences. While it may be difficult to recruit sufficient numbers of untreated, HIV positive individuals, future studies should include a larger HIV-negative group.

Our hypothesis was also based on the assumption that mtDNA levels would reflect overall cell death, regardless of mechanism. While DAMPs have been associated with both apoptosis and necrosis, their inflammatory activity often depends on the type of cell death. The initial study demonstrating the immunostimulatory activity of mtDNA was performed in trauma patients, where cell death is almost entirely due to necrosis [22]. It is not clear whether similar activity would be relevant in disease processes, like HIV, dominated by apoptosis. Interestingly, a report linking mtDNA-associated inflammation and heart failure suggests that only mitochondria that escape autophagy may stimulate pattern recognition receptors [28]. Perhaps regulated cell-death processes like HIV-associated apoptosis minimize the release of mtDNA and other inflammatory signals.

We performed additional analysis in our large cohort of long-term treated individuals. In the absence of ongoing HIV replication, which can have complex effects on end-organ function, important associations might be more easily observed. We found no clear effects of proximal CD4+ T cell count, self-reported CD4+ T cell count nadir, treatment regimen, or hepatitis C co-infection on mtDNA levels. We did, however observe a significant and unexpected negative correlation between age and plasma mtDNA. Plasma mtDNA levels decreased with age in this group but not in the larger cohort. The fact that we observed a stronger correlation in the HAART suppressed group as opposed to the elite controllers suggests that treatment could have a negative impact on mtDNA copy number. More work in large cohorts of uninfected persons is needed to clarify any important age-associated changes in mtDNA levels.

Many studies have found that nucleoside reverse transcriptase inhibitor toxicity can impact mitochondrial copy number, as measured in peripheral blood mononuclear cells [29], [30]. It is therefore interesting that we did not observe this effect in our measurement of free mtDNA in plasma. Because our assay measures only free or mitochondria-associated nucleic acid, our results should not be interpreted in the context of data on NRTI toxicity derived from peripheral blood mononuclear cells.

In summary, despite massive increases in apoptosis of T cells, gut epithelial cells, and other cell types in HIV infection, we found no evidence for an impact of HIV or its treatment on plasma DAMP levels. However, we did observe a somewhat counterintuitive decline in DAMP levels with age. Future studies should assess whether accumulation of apoptosis-resistant senescent cells with age results in decline in DAMP levels.
